# S11-4 Physical activity prescription in the municipality of Camarena in Castilla-La Mancha, Spain

**DOI:** 10.1093/eurpub/ckad133.056

**Published:** 2023-09-11

**Authors:** Susana Aznar, Alberto Dorado, Javier Ortega, Eduardo Sanz, Cristina Romero, Enrique Lizalde

**Affiliations:** PAFS Research Group, Faculty of Sports Sciences, University of Castilla-La Mancha, Spain; PAFS Research Group, Faculty of Sports Sciences, University of Castilla-La Mancha, Spain; PAFS Research Group, Faculty of Sports Sciences, University of Castilla-La Mancha, Spain; PAFS Research Group, Faculty of Sports Sciences, University of Castilla-La Mancha, Spain; PAFS Research Group, Faculty of Nursing, University of Castilla-La Mancha, Spain; High Council for Sports of Spain, Madrid, Spain

## Abstract

**Purpose:**

A pilot study of the Physical Activity (PA) Prescription (PAP) Plan in the municipality of Camarena (Castilla-La Mancha - CLM, Spain) was conducted to evaluate the incorporation of the exercise units which work together with General Practitioner (GP) to make “PA practice the easy option”. Target group is inactive people with or without chronic disease.

**Project or policy description:**

The High Council for Sports of Spain launched a national PAP plan developed by the creation of exercise units that will work together with PA referral (PAR) in all autonomic Communities in Spain. The plan is using digital tools to improve the accessibility and efficacy of the Community Sport and Health systems to provide PA for the inactive population with or without chronic disease. This investment is part of the Sports Sector Digitization Plan, one of the three axes of the Component 26, of the Recovery, Transformation and Resilience Plan, aimed at promoting the sports sector.

A pilot study in PAP was implemented in Camarena designed by PAFS research group from the University of CLM. The pilot study started with a local meeting including all agents in the community: local nurses and general practitioners (GP), all staff involved in the community PA and Sports offer, and town hall members.

The pilot study included: (i) developing a both-ways communication system between GP practice and the exercise unit, (ii) developing a Physical Fitness and Health assessment battery tool for all patients, (iii) developing individualized exercise programs using digital tools, (iv) analyzing all opportunities to be physically active at the community, (v) quantitative and qualitative evaluation analyses of the implementation of the PAP in Camarena. This pilot study aimed to be the seed to scale-up PAP plan in CLM region.

**Conclusions:**

The PAP plan had a good acceptance by the community of Camarena. The response rate of patients was high (85% of referred patients came to the exercise unit). Patients’ adherence to the exercise program was excellent. An educational plan is now developed as a policy-implication, for nurses, doctors, and PA educators in CLM, including all outcomes from PAP plan in Camarena.

